# Brownian diffusion of AMPA receptors is sufficient to explain fast onset of LTP

**DOI:** 10.1186/1752-0509-4-25

**Published:** 2010-03-16

**Authors:** Dominic P Tolle, Nicolas Le Novère

**Affiliations:** 1 Computational Neurobiology Group, EMBL-European Bioinformatics Institute, Wellcome Trust Genome Campus, Hinxton, Cambridge, CB10 1SD, UK

## Abstract

**Background:**

Long-Term Potentiation (LTP) of synapses is thought to be due in part to a change in AMPA Receptor trafficking leading to an increase in the number of AMPA Receptors at the synapse. LTP onset occurs within seconds after the induction signal. A particle-based stochastic simulation software is used to investigate the effect of Brownian diffusion of glutamate receptors on receptor incorporation into the synaptic specialisation and the time-course of LTP expression. The model of the dendritic spine includes receptors diffusing within the membrane, scaffold molecules within the synaptic specialisation capable of binding receptors and a molecular picket-fence surrounding the synaptic membrane area, all features found within the biological system.

**Results:**

During simulations, receptors accumulate rapidly at the post-synaptic density (PSD) from the extra-synaptic membrane under a number of biologically observed conditions. The time of half-saturation, *t*_1/2_, defined as the time-point at which half the available scaffold proteins are occupied with receptors, is found to be 710 ms. Different scaffold distributions are shown to have little effect on this time-course. Decreasing the probability of escape of receptors from the PSD domain, thus localising receptors closer to the scaffold proteins, substantially decreases *t*_1/2_. A decrease of escape probability from 1 to 0 brings about a non-linear decrease in *t*_1/2 _from 710 ms to 390 ms. Release-location of receptors within the spine is found to affect the initial rate of receptor incorporation. We simulate three possible sources of receptors: (i) receptors distributed within the spine extra-synaptic membrane; (ii) receptors from exocytotic vesicles released to the synaptic spine; and (iii) receptors entering the spine from the dendritic shaft through the spine neck. Receptors released from exocytotic vesicles initially accumulate faster than receptors released from the other two sources. A model of glutamate release and glutamate-receptor interaction shows that newly inserted receptors make a substantial contribution to a glutamate evoked response within the observed time-frame.

**Conclusions:**

Fast accumulation of AMPA Receptors is consistent with experimentally observed fast onset of LTP expression.

## Background

Fast excitatory synaptic transmission in the vertebrate brain is mediated by the *α*-amino-3-hydroxy-5-methyl-isoxazolepropionic-sensitive subtype of ionotropic glutamate receptors (AMPARs). These receptors are found enriched at the Post-Synaptic Density (PSD), a protein-rich, electron dense, layer located opposite the pre-synaptic active zone [1]. Far from being static entities, AMPARs undergo movement and trafficking by lateral diffusion within the membrane, as well as to and from intra-cellular stores by endo-/exocytosis [2-4]. The movement of AMPARs has implications for the maintenance of synaptic strength during resting state, for synapse formation during synaptogenesis, and for synaptic remodelling during synaptic plasticity [5].

Synaptic plasticity is the capacity of the synapse to alter the efficacy of its transmission. One of the best studied forms of synaptic plasticity is Long-Term Potentiation (LTP), an activity-driven long lasting increase in synaptic strength, considered to be one of the molecular bases of learning and memory [6,7]. LTP expression is thought to be due to the modulation of the conductance of AMPARs present at the synaptic specialisation [8,9], a change in AMPAR trafficking leading to an increase in the number of AMPARs at the synapse [10-12], or both. The increase in signal amplitude brought about by LTP is detectable within approximately 10 seconds following the LTP induction event [13] and, if caused by an increase in receptor number, has been estimated to involve only a small number of additional AMPARs [14]. The small window of time within which an increase in signal amplitude becomes detectable places constraints on the mechanism of LTP expression. The source of AMPAR molecules for incorporation into the PSD is one such constraint. Additional receptors are thought to come from intracellular stores which are exocytosed to the neuronal membrane [10]. However, the exact locus of exocytosis has been difficult to pinpoint, with previous experiments suggesting either a site peripheral to the PSD [4] or at the nerve cell body [15]. Recent experiments point to the locus being on the dendritic shaft, close to the spine, but not the spine itself [16], while other suggest that the receptors incorporated in the synapse come from the extra-synaptic membrane (ESM) of the spine [17]. No exocytosis directly to the synapse or indeed to the dendritic spine membrane has been shown.

These observations, in conjunction with the discovery that AMPARs diffuse by Brownian motion in the ESM [2], led to the suggestion that the ESM pool of AMPARs alone could act as the source for receptors during LTP [18]. Although the density of extra-synaptic receptors is small compared to synaptic receptors [19], the large area of ESM compared to synaptic membrane area gives rise to a large source of extra-synaptic receptors. In effect, the synapse acts as a diffusion-trap for the receptors within the ESM upon an LTP induction signal. Activity within a synapse, as well as an increase in intracellular calcium, as occurs during the early stages of LTP induction, have been shown to reduce the movement of AMPARs in the plasma membrane [2,20].

A number of previous models were designed to investigate the diffusion of AMPARs in the synaptic membrane [21-23]. Earnshaw and Bressloff used a two compartment ODE model of the spine to investigate the effect of various trafficking parameters, such as the rate of exocytosis and endocytosis and exchange of receptors from the PSD to the ESM, on number of receptors in the PSD over the timescale of minutes [21]. In a subsequent model, the authors gain insight into the diffusion of receptors along the dendrite, with spines acting as diffusion traps [22]. The model of Holcman and Triller uses a Markovian model to determine the steady state behaviour of the synapse, and to illustrate how synaptic strength can be maintained despite the dynamics of the receptors [23]. The authors further examine how modulation of the dendritic spine size affects the number of receptors over time scales of many seconds.

These models have either used ODE models or abstract representation of the synaptic specialization and operate on timescales of seconds to minutes. None of the models deal on the timescale of milliseconds or takes account of the microstructure of the spine and the relative positioning of the interacting components. Yet geometry and spatial parameters are important when dealing with the diffusion in the PSD [20,24]. Particle-based monte-carlo simulations have frequently been used in the past to study movement and aggregation of membrane receptors [25,26].

We use an in-house developed particle-based stochastic simulation software (see accompanying paper) to investigate the effect of Brownian diffusion of AMPARs on receptor incorporation into the synapse and the time-course of LTP expression. A model of the dendritic spine is detailed, including AMPARs in the ESM, scaffold molecules capable of binding AMPARs in the PSD and a molecular picket-fence surrounding the PSD. We use the software and model to show that the diffusion-trap model for LTP expression is compatible with the experimentally observed time-course of LTP. Diffusion and incorporation of AMPAR from the ESM is sufficient to explain the fast onset of LTP. We analyse the response of the system to alterations in some of the numerical parameters which influence the binding of receptors to scaffold molecules, such as the diffusion coefficient of AMPARs and the AMPAR/scaffold binding radius. As would be expected from a diffusion-reaction system, an increase in either the diffusion coefficient of AMPARs or the binding radius both lead to more rapid accumulation of AMPARs at the synapse. Increasing the number of scaffold elements relative to the number of AMPAR molecules additionally increases the rate of AMPAR capture. In contrast, changes in the distribution of AMPAR binding scaffold elements in the PSD were found to have little effect on the time-course of AMPAR capture. Furthermore, we evaluate the effect of confinement of AMPAR to a PSD micro-domain on receptor incorporation and find that confinement of the AMPARs to the PSD area increases the rate of AMPAR capture by the scaffold element, by trapping AMPARs in the vicinity of scaffold elements. Release location of AMPAR is also found to have an effect on the time-course of receptor capture.

## Results

All simulations are performed using *Meredys*, an in-house developed, particle-based stochastic simulation software. Models are described using an implementation of NeuroML [27]. *Meredys *uses Monte Carlo algorithms to simulate molecular diffusion and reaction in a bounded simulation volume. A detailed description of the software is found in an accompanying paper.

### Receptor incorporation into the PSD

**Figure 1:**
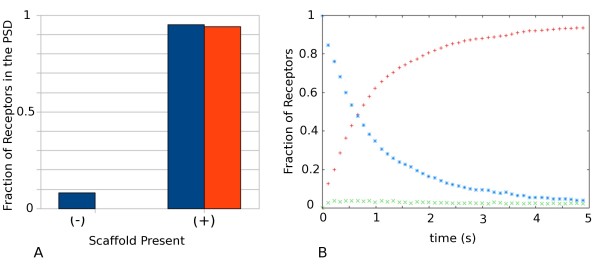
**Incorporation of Receptors into the PSD**. (a) Data show fraction of receptors present at the PSD following simulation of 5 seconds of diffusion in the absence (-) and presence (+) of AMPAR binding scaffold entities. Blue, total receptors; Red, receptors bound to scaffold. (b) Fraction of AMPARs bound, unbound in the PSD and in the ESM as a function of time. Red plus, bound; Green times, unbound in PSD; Blue stars, in ESM. Parameters used found in Table 1.

A diffusion-trap model for synaptic plasticity expression requires AMPARs to bind scaffold molecules at the PSD following random diffusion within the spine plasma membrane. Binding of the AMPARs to the scaffold effectively traps the AMPARs at the PSD, leading to an increase of receptor density at the synapse, and concomitant increase in post-synaptic signal amplitude, as is observed during LTP. The increase in the signal amplitude needs to occur within no more than 10 seconds [13]. The ESM contains a readily accessible pool of AMPARs [19], and receptors have been shown to exchange between the ESM and the PSD under resting conditions [2,28]. To test whether a sufficient number of AMPARs could accumulate at the PSD in the time-course allowed for LTP expression, we simulate diffusion of AMPARs in the model described above (see Table 1). The NeuroML files encoding the model can be found in the additional file 1. The simulations result in nearly complete capture of AMPARs after just 5 seconds of diffusion (Figure 1a). In comparison, a simulation run lacking scaffold elements shows 8% of AMPARs present within the PSD region of the dendritic spine, in good agreement with the total size of the PSD area (9% of the total spine area). The time-course of receptor binding to scaffold elements (Figure 1b) shows a fast depletion of AMPARs from the ESM, and a concomitant rapid accumulation of bound AMPARs in the PSD as AMPARs diffuse into the PSD area and bind to available scaffold anchors. As a measure of the speed of binding, we define the time of half-saturation, *t*_1/2_, as the time-point at which half the available scaffold binding proteins are occupied. In the case of the 'prototypical' reference model used (see Table 1) the time of half-saturation *t*_1/2 _= 710 ms. During the time span measured, the fraction of unbound receptors within the PSD area reaches a peak of approximately 0.04 and then declines steadily with the amount of free AMPARs available (Figure 1b, green curve). These results show that AMPARs can accumulate within the PSD from the pool of extra-synaptic receptors in the spine by diffusion within the time frame of LTP expression.

**Table 1:** Parameters.

Parameter	Abbreviation	Value	Reference
Spine Volume	*V*_*spine*_	0.5 *μ*m^3^	[55]
Spine Area	*A*_*spine*_	3.05 *μ*m^2^	calculated from above
Area of PSD	*A*_*PSD*_	0.27 *μ*m^2^	calculated from above
Radius of PSD	*r*_*PSD*_	295.4 nm	calculated from above
Area of ESM	*A*_*ESM*_	2.78 *μ*m^2^	calculated from above
Radius of AMPAR head particle	*r*_*AMPAR_ head*_	5 nm	
Radius of AMPAR tail particle	*r*_*AMPAR_tail*_	3 nm	
Radius of Scaffold particle	*r*_*scaffold*_	3 nm	
Diffusion Coefficient of AMPAR	*D*_*AMPAR*_	0.45 *μ*m^2^/s	[28]
Receptor density in ESM		20 *μ*m^-2^	[19]
Binding Radius	*σ*	0.5 nm	this article

Parameters used in the reference model

### Effect of Biophysical Parameters

**Figure 2:**
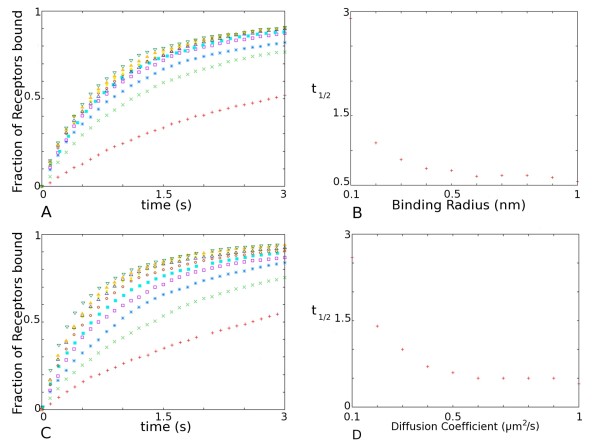
**Effect of Biophysical Parameters on time-course of Receptor capture**. (a) Time-course of receptor capture by the scaffold for a range of binding radii (*σ *= {0.1, 0.2, 0.3,...,1} nm). Red plus, 0.1; Green times, 0.2; Blue stars, 0.3; Purple square, 0.4; Cyan filled square, 0.5; Red circles, 0.6; Yellow bullets, 0.7; Blue up triangles, 0.8; Orange filled triangles, 0.9; Green down triangles, 1. (b) Time of half saturation as a function of binding radius. (c) Time-course of receptor capture by the scaffold for a range of diffusion coefficients (*D *= {0.1, 0.2, 0.3,...,1} *μm*^2^/*s*). Red +, 0.1; Green ×, 0.2; Blue *, 0.3; Purple squares, 0.4; Cyan filled squares, 0.5; Red circles, 0.6; Yellow bullets, 0.7; Blue triangles, 0.8; Orange filled triangles, 0.9; Green down triangles, 1. (d) Time of half saturation as a function of diffusion coefficient. Parameters used found in Table 1.

Capturing of ESM AMPARs by scaffold elements found within the PSD requires the receptors to diffuse across the ESM into the PSD to encounter scaffold molecules. The simulation software implements a bimolecular reaction algorithm designed by Andrews and Bray [29], which utilises the binding radius, *σ*, as the separation at which two reactants react (see accompanying paper). The binding radius is either user supplied, or determined from user supplied rate constants and the reacting molecules diffusion coefficients. The value of the diffusion coefficient of AMPARs and the size of the binding radius are therefore important parameters in determining the time-course of capture. In the absence of any information regarding the molecular identity of the scaffold anchor for AMPARs at the PSD, a range of binding radii is used and the effect on receptor incorporation is examined (Table 2). Figure 2a shows the rapid accumulation of AMPARs at the PSD for a range of binding radii (0.1 nm - 1 nm) following 5 seconds of simulated diffusion. An increase in binding radius (*σ*) gives rise to a decrease in *t*_1/2 _as AMPARs need to explore less area before coming into binding distance of a free scaffold molecule (Figure 2b).

**Table 2:** Reaction Rates 1.

**Reaction Rate (in Ms**^-1^**)**	Binding radius (in nm)
2550	0.1
20300	0.2
67300	0.3
154000	0.4
289000	0.5
473000	0.6
700000	0.7
963000	0.8
1253000	0.9
1590000	1

Reaction rates and binding radii for diffusion coefficient *D*_*AMPAR *_= 0.45 *μ*m^2^/s

Additionally, we investigate the effect of different magnitudes of AMPAR diffusion coefficients on the time-course of receptors incorporation (Figure 2c). The range of diffusion coefficients explored runs from 0.1 *μ*m^2^s^-1 ^to 1 *μ*m^2^s^-1 ^(using a 0.1 *μ*m^2^s^-1 ^increment) in accordance with values measured using single-molecule fluorescence microscopy [28] (Table 3). The diffusion coefficient for AMPARs is adjusted by adjusting the viscosity of the membrane environment [30]. An increase in the diffusion coefficient of AMPARs leads to a marked increase in the rate of receptor capture, as a higher diffusion coefficient allows a receptor to explore a larger area in less time. The time point of half saturation for the lowest diffusion coefficient it nearly 6.5 times the *t*_1/2 _for the highest diffusion coefficient (Figure 2d). Slower diffusing receptors tend to spend more time diffusing within the ESM before reaching available scaffold elements in the PSD. Although the diffusion coefficients of AMPAR receptors within the cell membrane can vary by an order of magnitude, accumulation of receptors in the PSD occurs within the time span measured for LTP expression for the range of experimentally determined diffusion coefficients.

**Table 3:** Reaction Rates 2.

Reaction Rate (in Ms^-1^)	Diffusion Coefficient (in *μ*m^2^/s)
185000	0.1
245000	0.2
271000	0.3
285000	0.4
293000	0.5
298000	0.6
301000	0.7
303000	0.8
306000	0.9
307000	1

Reaction rates and diffusion coefficient for binding radius *σ *= 0.5 nm

### Effect of Scaffold Distribution and Density

**Figure 3:**
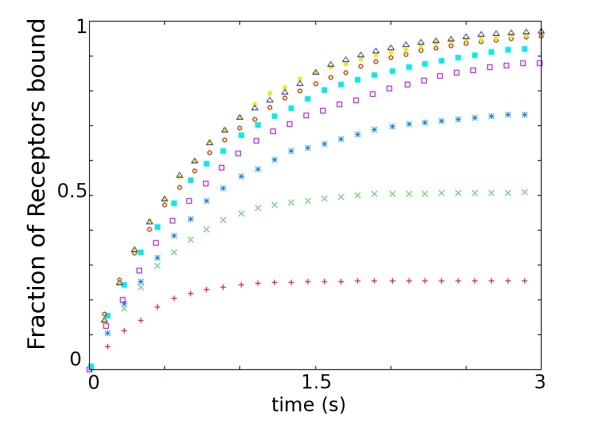
**Effect of the Ratio of Scaffold to AMPAR**. Fraction of receptors bound (of total possible bound) as a function of time for a range of scaffold/AMPAR ratios (*ρ *= {0.25, 0.5, 0.75,...,2}) Red plus, 0.25; Green times, 0.5; Blue stars, 0.75; Purple squares, 1.0; Cyan filled squares, 1.25; Red circles, 1.5; Yellow bullets, 1.75; Blue triangles, 2.0. Parameters used found in Table 1.

The molecular nature of the scaffold element responsible for anchoring AMPARs to the PSD is not yet known. Numerous potential candidates have been proposed, such as PSD95 (postsynaptic density protein 95 kDa), SAP97 (synapse-associated protein 97 kDa), GRIP (glutamate receptor interacting protein) and ABP (AMPA receptor binding protein)(see reviews [31,32]). Many of these are present in large quantities at the PSD [33]. The accumulation of AMPARs to the PSD depends on the availability of scaffold molecules capable of binding the receptors. To test the effect of an excess of both AMPARs in relation to binding sites, and scaffold in relation to AMPARs, we run a series of simulations with varying ratios of scaffold to AMPAR molecules. In each series, the number of AMPARs is kept constant, while the number of scaffold molecules is changed relative to the number of AMPARs. Figure 3 shows the time-course of receptor capture for a range of different scaffold-to-AMPAR ratios. Excess of receptors over scaffold elements (ratio *<*1), and excess of scaffold over receptors (ratio *>*1), both lead to faster accumulation of receptors at the PSD compared to when both entity types are present in equal amount (ratio = 1). Overabundance of the mobile element (i.e. the receptor) gives rise to the fastest accumulation time.

**Figure 4:**
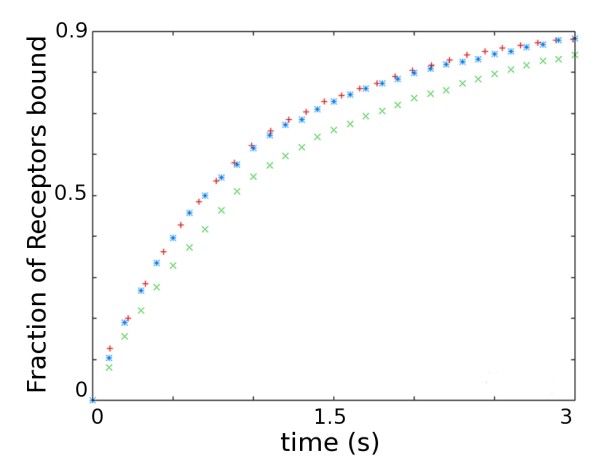
**Effect of Scaffold distribution on time-course of Receptor capture**. Time-course of AMPAR capture to the PSD for different distributions of scaffold molecules. Red plus, uniform; Green times, annular; Blue stars, patch. Parameters used found in Table 1.

**Figure 5:**
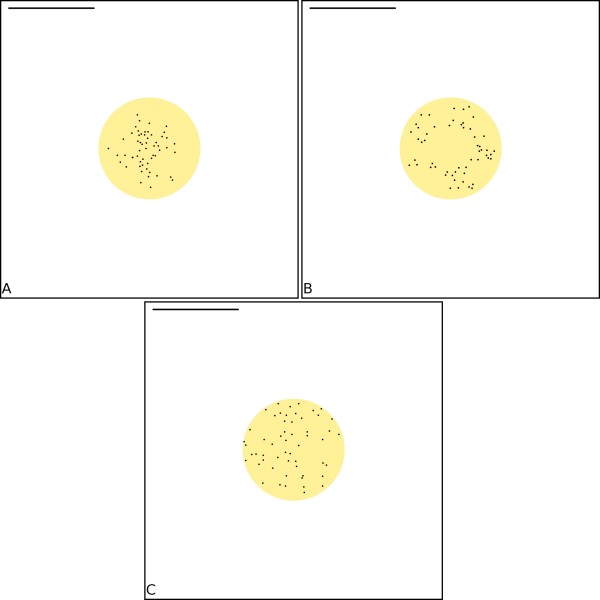
**Schematic representation of model spine and distribution of scaffold elements**. (a) Scaffold element position within the PSD drawn from a Uniform distribution (see 'Materials and Methods' for details). (b) Scaffold element position within the PSD drawn from a Annular distribution (see 'Materials and Methods' for details). (c) Scaffold element position within the PSD drawn from a Patch distribution (see 'Materials and Methods' for details). Scale bar is 500 nm.

Immunogold labelling has determined a number of possible distributions for AMPARs at the PSD [34-36]. We test the effect of scaffold binding distribution on the time-course of AMPAR capture (Figure 4). Three different distributions are tested: uniform, annular, patch (see Figure 5 and 'Methods'). Change in distribution of scaffold molecules within the PSD has little effect on the time-course of receptor capture (Figure 4), although the annular distribution displayed a slightly slower rate at later times, past the time point of half saturation. This is most likely due to the larger number of receptors closer to the edge of the PSD domain in the uniform and patch distribution compared to the annular distriution. Time points of half saturation for the uniform, annular and patch distribution are 710 ms, 880 ms and 700 ms respectively.

### Effect of Confinement

**Figure 6:**
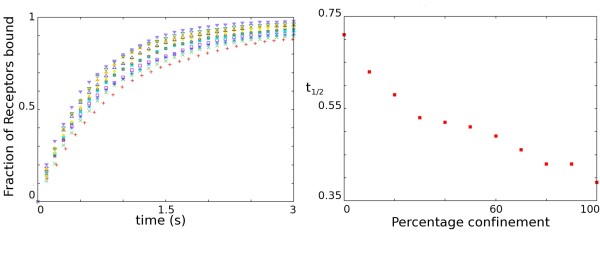
**Effect of Confinement on time-course of Receptor capture**. (a) Time-course of receptor capture by the scaffold for a range of reflection probabilities (*P *(*Reflection*) = {0.0, 0.1, 0.2,...,1}). Red plus, 0; Green times, 0.1; Blue stars, 0.2; Purple squares, 0.3; Cyan filled squares, 0.4; Red circles, 0.5; Yellow bullets, 0.6; Blue triangle, 0.7; Orange filled triangles, 0.8;Green down triangles, 0.9; Blue filled down triangles, 1. (b) Time of half saturation as a function of reflection probability. Parameters used found in Table 1.

Diffusion of AMPARs within the post-synaptic specialisation is not unrestricted but occurs in a confined area [24]. By locally trapping AMPARs within the vicinity of the AMPAR binding scaffold molecules it is conceivable that the rate of receptor capture to scaffold proteins is increased. We model this confinement by changing the boundary condition for PSD to ESM from an open boundary to a partially reflective boundary. The change from open boundary to partially reflective boundary causes each AMPAR crossing the PSD to ESM boundary to have a probability of being reflected back into the PSD rather than entering the ESM. The results for simulations implementing a range of confinement parameter values (*P *(*Reflection*) equals 0 to 1) and the effect on the time-course of receptor capture are displayed in Figure 6a. Confinement leads to accumulation of AMPARs over a shorter time period. The *t*_1/2 _decreases from 710 ms to 390 ms as *P *(*Reflection*) increases from 0 to 1 (Figure 6b). An increase in boundary reflection prevents AMPARs from diffusing out of the PSD again once they enter the PSD area. Although the effect is low, modulation of AMPAR confinement within the PSD does affect the time-course of receptor capture to the PSD.

### Release location of AMPARs

**Figure 7:**
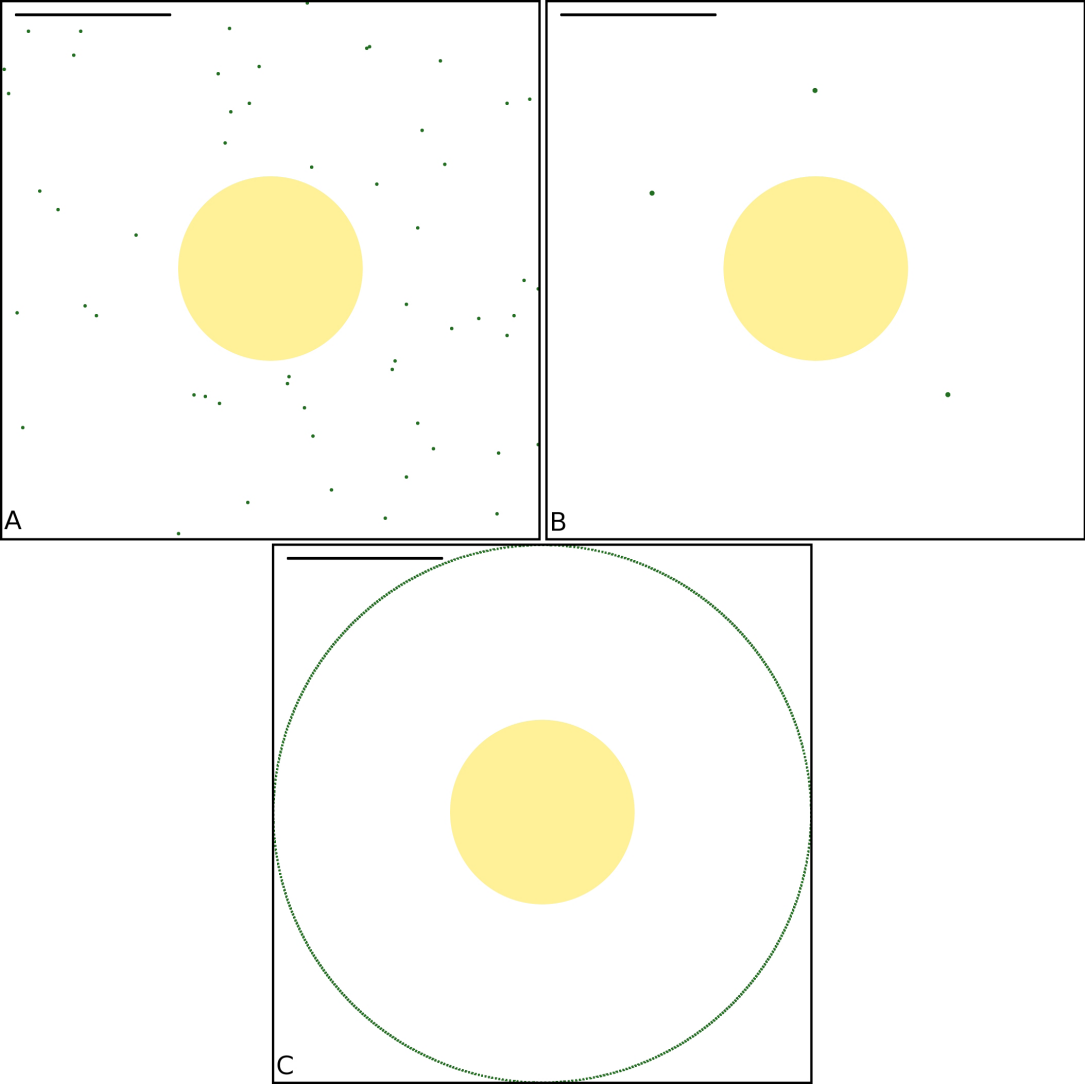
**Schematic representation of model spine and release location of AMPAR**. (a) AMPAR release location within the ESM drawn from a Uniform distribution (see 'Materials and Methods' for details). (b) AMPAR release location within the ESM from 3 point-sources (583.95 nm from the PSD centre) (see 'Materials and Methods' for details). (c) AMPAR release location within the ESM from an annulus around the PSD (872.5 nm from the PSD centre) (see 'Materials and Methods' for details). Scale bar is 500 nm.

**Figure 8:**
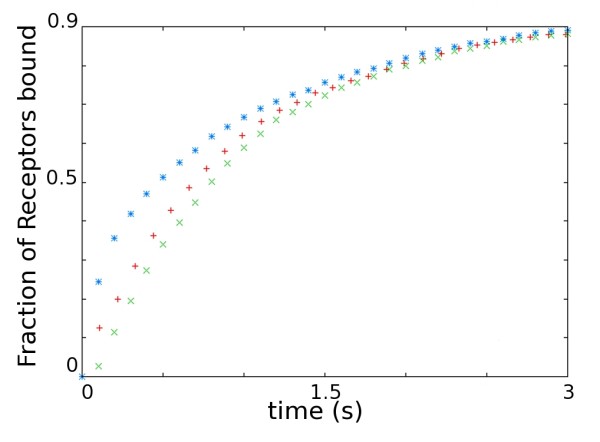
**Effect of release location of AMPAR on time-course of capture**. Time-course of AMPAR capture to the PSD for different release locations for AMPAR molecules. Red plus, uniform; Green times, annular; Blue stars, point-source. Parameters used found in Table 1.

Intracellular pools of receptors, exocytosed during LTP induction, have also been proposed as the source of AMPARs for LTP expression. The site of AMPAR exocytosis has not been determined yet. We model both the appearance of AMPARs by exocytosis peripheral to the PSD and from the spine neck by changing the starting location of AMPARs (Figure 7). Exocytosis peripheral to the PSD is modelled by releasing 3 batches of AMPARs (containing 18, 18 and 19 receptors respectively), from 3 point sources a distance of 583.95 nm from the PSD centre, corresponding to a point half way between the edge of the PSD and the point of contact with the spine neck (Figure 7b). The entrance of AMPARs into the spine via the spine neck is modelled by placing the AMPARs uniformly on an annulus 872.5 nm from the PSD centre, corresponding the point of contact with the spine neck (Figure 7c). The effect of the different release locations can be seen in Figure 8. The initial rate of receptor capture for point released AMPAR is higher than both the receptors released uniformly in the ESM and receptors released in an annulus around the PSD. This is likely due to the differences in the initial distances of the released AMPARs from the PSD.

### The Model of Glutamate Signalling

**Figure 9:**
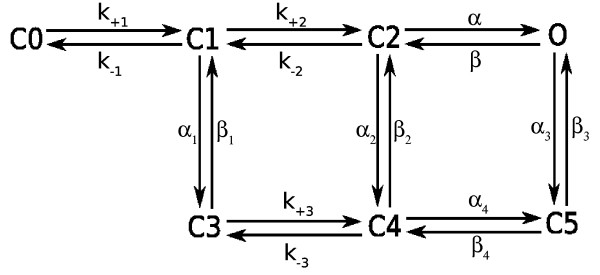
**Kinetic scheme of the AMPAR**. Rate constants used were determined by Jonas *et al*. [37] and are found in Table 5. Naming convention of the states match those jound in Jonas *et al*. Two glutamate molecules need to be bound for the channel to switch to the open state (C2 to O). Desensitization can occur from the single-bound, closed-channel; double-bound, closed-channel; or double-bound, open channel state (C3, C4, C5).

To measure the effect of glutamate release on post-synaptic receptors, a kinetic model of AMPARs is required, detailing binding of glutamate, as well as channel opening and channel desensitisation. The NeuroML files encoding the model can be found in the additional file 2. The kinetic scheme of the AMPAR channel and accompanying rate constants (Figure 9) are taken from Jonas *et al*. [37]. The kinetic constants determined by Jonas *et al*. are based on experiments performed at 25° Celsius. Values are brought to their 37° Celsius equivalent by applying a *Q*_10 _temperature coefficient of 3.0 as described in Wahl *et al*. [38](see Equation 1).(1)

Where *R *is the rate, and *T *is the temperature in Celsius.

**Figure 10:**
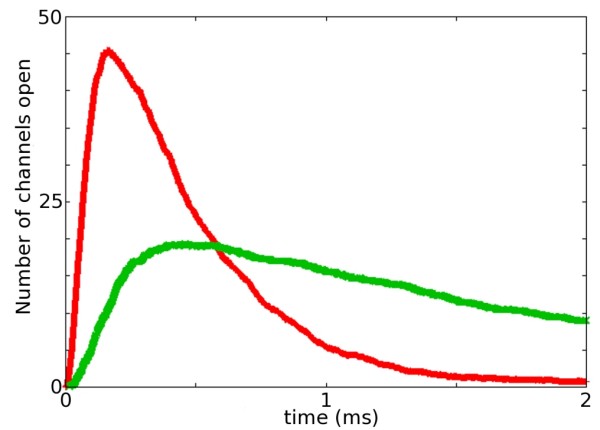
**Comparison of temperature adjusted rate constants with original rate constants**. Signal amplitude, rise time, and decay are increased for the response when the temperature adjusted rate constants are used. Green, Rate constants of Jonas *et al*.; Red, temperature adjusted rate constants

Figure 10 compares the time course of AMPAR channel opening following a release of glutamate using both sets of kinetic constants. An ensemble average of a signalling simulation series is used to calculate the time-course of receptor opening. A total of 4000 glutamate molecules is released at time zero and allowed to diffuse across the synaptic cleft [39]. The number of open AMPARs is measured at each time step. The time course of the ensemble average using the kinetic constants reported in Jonas *et al*. (green trace) displays a 10% - 90% rise time of 0.24 ms (20% - 80% rise time 0.15 ms) and a *P*_*o,max *_of 0.24 similar to results reported in previous models [38]. In comparison, the time course of the ensemble average using the temperature adjusted kinetic constants show a 10% - 90% rise time of 0.09 ms (20% - 80% rise time 0.06 ms) and a *P*_*o,max *_of 0.57, also is in agreement with previous models [14,38] as well as experimental measurements taken close to body temperature [40].

These results show that the model can simulate glutamate signalling effectively, comparing well with published results for both previous models and laboratory experiments.

### AMPA Receptor Capture during Glutamate Release

The above results show that diffusion and incorporation of AMPAR can rapidly increase the number of receptors within the PSD. However, the early incorporation of receptors may not immediately translate into an increase in excitatory post-synaptic current (EPSC) strength. It has been pointed out that the majority of receptors activated during an EPSC are done so by an initial 'spike' of glutamate concentration close to the glutamate release site [14]. In addition, spacing between receptors has a marked effect on the height of the signal - as the spacing between receptors increases, the height of the response drops [38]. It is expected that the accumulation of receptors occurs first at the periphery of the PSD, as the scaffold elements present there are first encountered by a diffusing AMPAR upon reaching the synapse. As a consequence, the effect of this incorporation on the EPSC needs to be further investigated.

**Figure 11:**
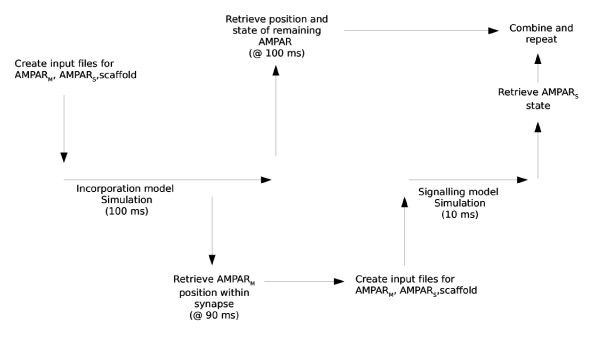
**Outline of compound model**. The model includes both simulation of receptor incorporation and simulation of glutamate signalling.

In order to see how the accumulation of receptors over time affects the receptor signal elucidated by glutamate a compound model is created comprising AMPAR incorporation into the PSD with glutamate release and binding to synaptic AMPAR. Figure 11 shows an overview of the compound model. Firstly, an incorporation model simulation is run simulating 100 ms of receptor diffusion by Brownian motion within the dendritic membrane. The model includes mobile receptors (N = 55), starting uniformly distributed in the ESM, static receptors (N = 100), uniformly distributed within the PSD, and scaffold elements (N = 55), also uniformly distributed in the PSD. Following the simulation, the state (i.e. position and feature state) of the mobile receptors within the simulation is taken at the time point following 90 ms of diffusion. The states of these receptors, in addition to the states of the static receptors and scaffold elements, are used as input for a signalling model simulation. If a mobile receptor is found bound to a scaffold element, the receptor is added to the immobile pool of receptors and removed from the pool of mobile receptors. The affected scaffold element is removed from the pool of scaffold elements.

The signalling model simulation simulates 10 ms of the glutamate signal. At the end of the signalling simulation, the state of the synaptic receptors is noted and merged with the state of the remaining receptors, taken from the output at the end of the preceding incorporation simulation. The whole procedure is then repeated.

**Figure 12:**
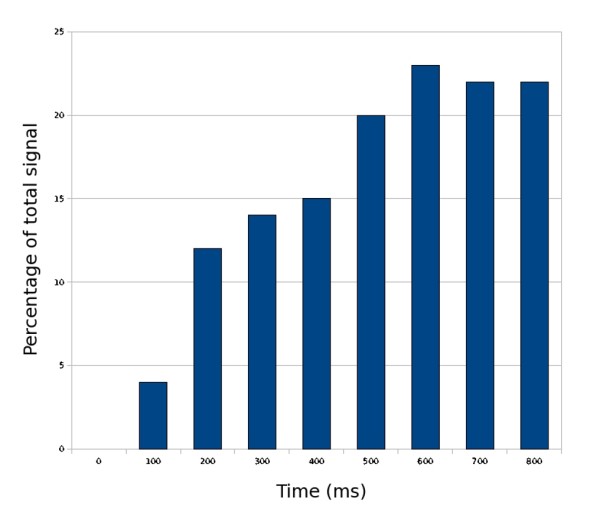
**Percentage contribution of new receptors to synaptic signal**. An ensemble average of a simulation series is used to calculate the cumulative percentage contribution of newly incorporated receptors to the synaptic signal. Time points indicate the end-time for the glutamate release simulation (each simulation was run for 10 ms). The number of receptors open at the peak response during the 10 ms measurement was used to calculate the percentage.

Figure 12 shows percentage contribution of newly incorporated receptors to the glutamate evoked signal at 100 ms intervals. As more new receptors are incorporated to the synapse over time, the contribution of the newly incorporated receptors to the glutamate signal increases. By the time of half-saturation, between 700-800 ms, the new receptors account for over 20% of the glutamate signal. Table 4 shows the average lateral distance of newly incorporated receptors which participate in signal generation from the glutamate release site. The average distance of newly incorporated receptors decreases as more receptors are incorporated to the synapse. This is presumably because binding sites closer to the edge of the PSD are first to capture mobile receptors.

**Table 4:** AMPAR distances.

Time point	Mean distance
100 ms	223.4 +/- 63.29 nm
200 ms	224.5 +/- 60.77 nm
300 ms	221.5 +/- 63.35 nm
400 ms	220.9 +/- 63.35 nm
500 ms	220.1 +/- 62.9 nm
600 ms	219.2 +/- 64.18 nm
700 ms	217.9 +/- 64.63 nm
800 ms	214.7 +/- 67.08 nm

Lateral distance of newly incorporated AMPAR from glutamate release site

## Discussion and Conclusions

We present a biophysical realistic model to investigate the effect of AMPAR movement in the post-synaptic membrane during the initial phase of LTP expression. The effect of AMPAR diffusion parameters, and PSD scaffold composition and geometry, on the incorporation of receptors into the PSD is analysed. Further, the effect of receptor incorporation into the synapse on the post-synaptic signal are examined. The model system incorporates AMPARs diffusing in the membrane, scaffold proteins, capable of binding AMPARs, distributed within the PSD, and glutamate release from postsynaptic stores and interacting with membrane receptors. Knowledge of the distribution of receptors within the synaptic membrane [35,36] was used in the construction of the models. The diffusive behaviour of AMPARs, as observed in particle-tracking experiments [28], was also incorporated in the models. None of the models of AMPAR diffusion to date have probed the effect of the different distributions of scaffold elements on the incorporation of AMPARs at the synapse. Yet, theoretical models have shown that the placement of traps can affect the rate of diffusion-limited processes substantially [41].

The model and accompanying simulation results support the hypothesis that AMPARs can come from the pool of extrasynaptic receptors to cause LTP expression within the allotted time and by random diffusion alone. For the range of measured diffusion coefficient and a range of binding radii, AM-PARs can accumulate within the PSD within the time frame of LTP expression [13]. The response of the model to changes in the ratio of scaffold elements to AMPARs, different initial distributions of both scaffold elements within the PSD and AMPARs within the ESM, and a change in the confinement of AMPARs to the PSD area is analysed. The time of half-saturation, *t*_1/2_, was used as a measure of the speed of binding. It is dependent on the diffusion coefficient of the receptors, the binding radius of the receptor-scaffold interaction, the number of interacting components, as well as the average initial distance of the receptors from the scaffold elements. This distance, in turn, is dependent on the receptor and the scaffold initial distributions.

AMPAR movement in the PSD is thought to be affected mainly by two factors: (i) interaction with scaffold molecules, and (ii) entrance/exit rates of receptors to/from the PSD. The exact nature of the protein responsible for anchoring AMPARs to the PSD during LTP induction remains elusive. The search is made more complicated by the difficulty in differentiating between molecules responsible for targeting AMPARs to the PSD as compared to molecules responsible for maintaining AMPARs at the PSD [5]. Either may also be different for different AMPAR subtypes [42], or may not even bind to AMPARs at all, but to their auxiliary proteins instead [43,44]. As a consequence, it is difficult to estimate the affinity of AMPARs for scaffold elements, or the density and distribution of scaffold proteins in the PSD. Several plausible models are considered in this study.

The model system uses the binding radius, the maximum distance two molecules can approach each other before reacting, as a measure of the affinity of AMPARs for the scaffold binding molecules, as detailed by Andrews and Bray [29]. The binding radius is derived from Smoluchowski's theory for reaction rates [45], and in the algorithm is calculated from the reactants diffusion coefficients, the reactions experimental reaction rate, and the Brownian Dynamics algorithms step length. For diffusion limited reactions this is equal to the sum of the molecular radii of the interacting components [29]. As the exact nature of the protein-protein interaction trapping AMPARs at the synapse is unknown and experimental reaction rates are missing, a range of possible binding radii is tested. All the radii fit into a biologically meaningful range. The results indicate that for all the binding radii tested incorporation still proceeds rapidly within the seconds range (figure 1a). Whether, and how, an LTP induction stimulus can rapidly regulate the anchor sites remains to be determined. A likely model is that anchor molecules are already present at the synapse, and "activated" by the rise in Ca^2+ ^brought about by Ca^2+ ^influx through the NMDAR. Such a model would be consistent with the observed decrease in receptor mobility following Ca^2+^uncaging [2].

Rates of reactions in the above system also depend on the diffusion coefficient of the reacting entities. The effect of the AMPAR diffusion coefficient on the time course of receptor incorporation are seen in figure 1c. Factors influencing the diffusion coefficient of a protein in a membrane include the radius of the proteins membrane spanning region and the viscosity of the membrane among other factors. A number of diffusion coefficients have been measured for AMPARs within the neuronal plasma membrane using single-molecule fluorescent microscopy [28], possibly reflecting the heterogeneity of the lipid environment in the neuronal membrane [46], as well as the association of AMPARs with other membrane spanning proteins [44].

A number of possible distributions for AMPARs at the PSD, ranging from uniform [34] to annular [35] or patchy [36], have been determined. The exact ultrastructure of the PSD has not been determined, but presumably the observed distribution of AMPARs reveals the underlying distribution of AMPAR binding scaffold proteins in the PSD. As the placement of traps in different spatial arrangements can have a substantial effect on the rate of diffusion-limited processes such as the diffusion to capture [41], all of the above distributions were tested. Distribution of scaffold molecules within the PSD has little effect on the time course of receptor capture (Figure 5). Although the annular distribution displayed a slightly slower rate after an initial period, this is most likely due to the larger number of receptors closer to the edge of the PSD domain in the uniform and patch distribution compared to the annular distribution. Regardless of distribution, scaffold elements do saturate rapidly.

In the model, corralled diffusion within the PSD area is examined. The restriction to diffusion is uni-directional only, with AMPARs allowed to enter the PSD area but restricted in exit from the PSD. This restriction localises the AMPARs to the PSD and hence in the vicinity of the scaffold molecules. The effect of the PSD corral on the incorporation of receptors is noticeable for the duration of the measurements, with a more secure corral leading to an increase in the initial rate of receptor incorporation, as well as a lower *t*_1/2_. Whether a similar mechanism is utilised *in vivo *remains to be seen. Receptors have been shown to undergo confined diffusion [24,28] once they enter the synapse. Even the synapse itself appears to contain sub-domains [20]. The exact reason for this is as yet unknown, though models suggest that synaptic strength can vary strongly depending on the correlation of post-synaptic receptor placement and presynaptic glutamate release [14,47]. It should be noted that the experimental data for AMPAR diffusion does not allow for the differentiation between confined diffusion and obstacle-impeded diffusion [48]. Although the above model assumes diffusion within a corral, both processes probably influence synaptic AMPAR diffusion *in vivo*.

The source of the AMPARs required for LTP expression may be receptors present in intracellular stores [10,49]. However, the locus of receptor exocytosis has not yet been determined. Various methods used have placed the location of exocytosis into the spine but peripheral to the PSD [4], in the dendrite close to the spine but not the spine itself [16], or at the nerve-cell body [15]. All of these scenarios require the AMPARs to translocate to the PSD. The latter two depend on AMPARs entering the spine through the spine neck. If the spine neck can act as a diffusion barrier [50] then this may require the utilization of motor proteins accounting for the observation that myosin Va is required for AMPAR insertion into the synapse [51]. In either case, the release location of AMPARs affects the time-course of receptor incorporation. Exocytosis closer to the PSD greatly increases the initial rate of receptor capture to the PSD. The rates for the three release distributions tested converge as the remaining receptors in each simulation series reach diffusional equilibrium.

The contribution of newly incorporated receptors to the glutamate evoked signal is measured. It has been proposed that only a few extra open AMPARs may be necessary to increase the amplitude of the signal for LTP [14]. The same model suggests that 80% of the current is carried by channels in a 240 nm diameter region around the release site. The model presented shows that receptors captured to the synapse following a diffusion/trap model are first incorporated at peripheral binding sites within the PSD, assuming uniformly distributed anchor molecules. It is conceivable that the sequestering of receptors by binding sites at the periphery of the PSD could lead to insufficient proximity of newly acquired receptors to the glutamate release site for the receptors to participate in the signal. However the model shows that distant receptors still contribute to the glutamate signal. In addition, the model suggests that newly acquired receptors contribute to the signal very early on in the incorporation process. This observation is consistent with the idea of extrasynaptic receptors acting as the source of new receptors during LTP expression.

Many questions remain to be answered, and as more data becomes available, the details of the model will change and be refined. The kinetics of the interaction of receptors with scaffold proteins should be further investigated. Anomalous diffusion has been observed for receptors diffiusing in the synapse, and attributed to confinement [2,5]. However the causes of anomalous diffusion can be many and, as previously mentioned, the available data does not point conclusively to diffusion within a confined domain [48]. Transient interactions can lead to similar behaviour. Research suggests that the PSD itself may be divided into specific sub-domains which impact on the glutamate evoked signal [20]. This division of the synapse into subcompartments requires more examination. Especially the organisation of receptors in a sub-domain on the EPSC, how receptor concentrations can be controlled at the level of the sub-domain, and the effect of sub-domain correlation with the glutamate release site on the EPSC need to be addressed. Mobility of receptors within sub-domain and exchange between sub-domains, as well as exit and entrance from synapse are clearly factors affecting the incorporation of receptors into the synapse and the resulting increase in glutamate evoked current.

## Methods

### Model of the dendritic spine

The model used to describe the receptor movements in the dendritic spine includes the compartmentalisation of the dendritic spine plasma membrane into distinct membrane domains, diffusion of receptors within the plasma membrane, and the presence of scaffold molecules in the synaptic area capable of binding the receptors. The effect of changing various parameters on AMPAR accumulation at the PSD are investigated in this study. What follows is the description of an incorporation reference model used as the prototype for the subsequent construction of specific models. The various parameter values used in the reference model are given in Table 1.

The dendritic spine has been shown to exhibit only slow and limited diffusional exchange of surface receptors with the dendritic shaft [50]. To accommodate this observations, we model the spine as a self-contained diffusion compartment corresponding to a sphere of the same volume as a large dendritic spine (see Table 1). Boundary interactions for receptors with the simulation volume boundaries are 100% toroidal, effectively simulating a sphere. That is, receptors which diffuse across the simulations volume boundary are translated across the simulation volume, 'emerging' from the opposing simulation volume boundary. Viscosity of the membrane is chosen such that the diffusion constant for receptors, *D*, matches those observed in the biological system [28]. The total surface area of the dendritic spine plasma membrane, *A*_*spine*_, is calculated from experimentally measured values of the dendritic spine volume according to equation (2):(2)

The plasma membrane of the synaptic spine is modelled as a square with a surface area, *A*_*spine*_. The two membrane compartments that comprise the plasma membrane of the dendritic spine are the ESM and the synaptic plasma membrane corresponding to the PSD.

The PSD region of the synaptic spine is represented as a circular membrane domain with radius *r*_*PSD *_and surface area *A*_*PSD*_. Both the boundary conditions for molecules crossing from the PSD into the ESM and for molecules crossing from the ESM into the PSD are defined as open. AMPARs can traverse freely into and out of the PSD. The PSD is placed into the centre of the simulated plasma membrane, the centre of the PSD membrane domain being located at the simulation volume origin of coordinate (0,0,0). It has been estimated that the PSD occupies approximately 9% of the area of the synaptic spine membrane [52]. The surface area of the ESM is determined by(3)

### Molecules within the membrane

AMPAR entities are embedded in the membrane where they are allowed to diffuse freely. The density of AMPARs in the ESM is taken from values reported in the literature [19]. A cytoplasmic tail part allows AMPARs to interact with the scaffold entities located below the plasma membrane. Scaffold molecules are represented as separate, static entities. Scaffold entities are placed just below the PSD membrane domain to allow interaction with the tail region of receptor entities. The molecular nature of the anchoring site for AMPARs at the PSD is still not determined, and may well depend on the state of the individual synapse, as well as on the subtype of AMPAR [31,32]. Since the identity of the AMPAR binding scaffold is not known there are no experimentally observed values for the density of the scaffold elements within the PSD. We investigate the effect of changing scaffold density. For the incorporation reference model, however, we assume that the number of anchors is equal to the number of free AMPARs in the ESM.

**Figure 13:**
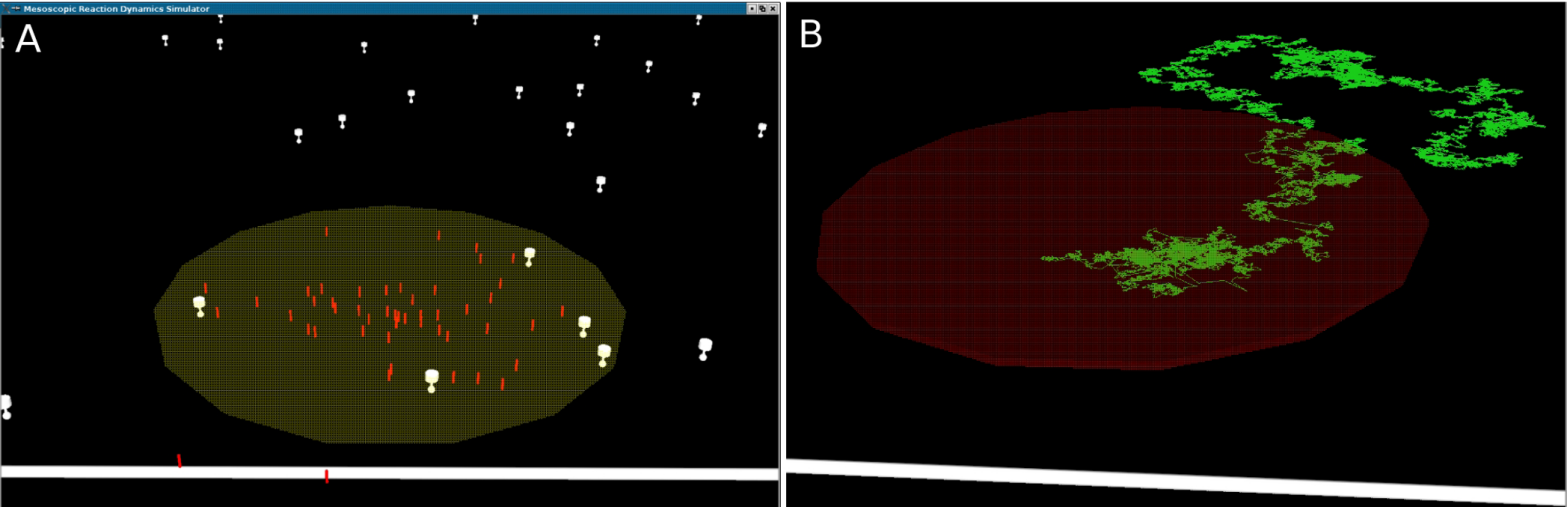
**Screenshot of *Meredys *and trace of receptor**. (a) Screenshot of a *Meredys *simulation. The plane of the membrane is viewed at a 45 degree angle. White receptor entities diffuse in the membrane. Red scaffold entities are distributed within the PSD microdomain (highlighted in yellow). (b) Example trace of individual AMPAR receptors displaying unrestricted Brownian diffusion. The PSD area is highlighted in red.

We are interested in the accumulation of AMPARs from the ESM to the PSD. Therefore receptors and scaffold elements do not start homogeneously distributed throughout the model membrane. Receptors are randomly placed within the ESM area of the membrane with coordinates drawn from a uniform distribution. The scaffold elements are randomly distributed within the PSD region of the synaptic spine with coordinates drawn from a uniform distribution. The model system consists of a limited number of adsorbers (scaffold anchors) and adsorbates (AMPARs), with both populations starting in two distinct domains (i.e non-homogeneous) and only the receptors allowed to diffuse in 2 dimensions (Figure 13). Additionally, reactions between adsorber and adsorbate remove both entities from the system.

**Figure 14:**
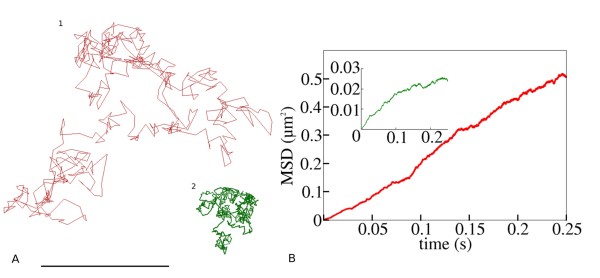
**Examples of AMPAR movement within the model system**. (a) Example trajectories of individual AMPAR receptors displaying unrestricted Brownian diffusion and diffusion in a confined area. Scale bar is 500 nm. Red trace, free diffusion; Green trace, confined diffusion in area of 300 nm diameter. (b) Plot of MSD versus time.

Within the extra-synaptic membrane, the diffusion of AMPAR has been shown to be unrestricted and Brownian in nature [2]. AMPAR diffusion within synaptic regions appears to occur in a confined region [24,28]. By adjusting the boundary condition for exit from the PSD membrane domain, from open to reflective, our model replicates this behaviour. Figure 14 demonstrates the reproduction of the two types of diffusion behaviours observed for synaptic AMPARs in our model system. Example traces are shown (Figure 14a) and the mean-squared displacement (MSD) of all the receptors in the simulation series is plotted against simulated time (Figure 14b). A simulation series of freely diffusing receptors in the spine membrane lacking AMPAR binding scaffold molecules and membrane domain corrals (red trace/plot in Figure 14) yields a linear dependency of the MSD on time, characteristic of unrestricted Brownian motion, and described by equation (4).(4)

Where *D *is the diffusion coefficient of AMPARs and *t *is time. Conversely, simulations with receptors surrounded by a circular corral of 300 nm radius with perfect boundaries (green trace/plot) and plotting MSD versus time displays levelling off of the plot, indicative of diffusion within a confined space. Phenomenologically equivalent behaviour has been observed in experimental systems [28].

### Distribution of Molecules in the Membrane

Each scaffold entity initial placement in the PSD was determined as follows:

#### Uniform Distribution

Polar coordinates for the position of the entity in the simulation volume where created by drawing two random numbers, *R *and *ϕ *from U(0,1) and U(0,2*π*), respectively and transformed into Cartesian coordinates by *x *= *cos*(*ϕ*) ** *radius*_*psd *_and *y *= *sin*(*ϕ*) * * *radius*_*psd*._

#### Annular Distribution

The PSD disk was divided into 5 concentric circles each of thickness . Each segment had a probability associated with it of a receptor being placed within it determined from the experimental data of Kharazia & Weinberg [35]. The scaffold entities are placed uniformly (see above) within each segment.

#### Patch Distribution

The PSD was composed of 5 disks of radius 96 nm, corresponding to the confinement radius measured in active synapses [20], arranged as a pentagon, with the centres of the disks 194.4 nm from the centre of the PSD. Receptors were placed as for the Uniform distribution above. Receptors that did not fall into any of the 5 disks were replaced.

AMPAR entity initial placement in the ESM area was determined as follows:

#### Uniform Source

Each Entity Cartesian coordinates were determined by drawing *X *and *Y *from U(-, ). The distance of (*x,y*) from the origin was calculated and if found to be less than *radius*_*PSD*_, the point was discarded and a new pair of random numbers created.

#### Annular Source

Polar coordinates for the position of the each entity in the simulation volume where created by drawing one random number, *ϕ *from U(0,2*π*). Coordinates where transformed into Cartesian coordinates by *x *= *cos*(*ϕ*) * d_*full *_and *y *= *sin*(*ϕ*) * d_*full *_Where *d*_*full *_is 872.5 nm from the PSD centre, corresponding the point of contact with the spine neck.

#### Point Source

Three point sources were randomly determined by drawing one random number, *ϕ *from U(0,2*π*). Coordinates were transformed into Cartesian coordinates by *x *= *cos*(*ϕ*) * d_*half *_and *y *= *sin*(*ϕ*) * d_*half *_Where *d*_*half *_is 583.95 nm from the PSD centre, corresponding to a point half way between the edge of the PSD and the point of contact with the spine neck. The first two points determined the initial position of 18 AMPAR and the last point determined the position of 19 AMPAR.

### The Model of Glutamate Signalling

**Figure 15:**
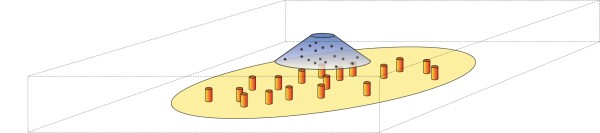
**Schematic of the signalling model**. The model includes the PSD (yellow circle), a glutamate release site (blue cone) and AMPARs distributed within the PSD (orange cylinders). The length and depth of the cuboid is 700 nm and the height is 22.5 nm. AMPARs (*N *= 100) are uniformly distributed within the PSD (*r *= 295.4 nm). Glutamate molecules (*N *= 4000) are released from a point source opposite the centre of the PSD.

The model used to describe glutamate signalling within the synapse includes the glutamate release site, the synaptic cleft, and the postsynaptic membrane including AMPARs (Figure 15). The various parameter values used in the signalling model are given in Table 5. The synapse is modelled as a cuboid of length 700 nm, depth 700 nm and height 22.5 nm. The 'floor' of the cuboid corresponds to the postsynaptic membrane area, and the 'ceiling' of the cuboid corresponds to the presynaptic bouton. The volume of the cuboid represents the synaptic cleft. Glutamate molecules are released from a point source 1 nm below the centre point of the cuboid ceiling. Previous models have shown that point-source release of glutamate is a good model for the release of glutamate through a fusion pore [14]. Glutamate molecule boundary interaction with the 'floor' and 'ceiling' of the simulation volume is reflective. The remaining simulation volume walls follow absorbing boundary conditions, simulating diffusion of glutamate molecules out of the synaptic cleft and subsequent absorption by surrounding cells. Diffusion out of the synaptic cleft appears to be the main mechanism of glutamate removal [38]. The postsynaptic membrane area contains a circular membrane domain demarcating the PSD. AMPAR entities are randomly distributed within the PSD region with coordinates drawn from a uniform distribution. For the reference model, the number of AMPARs present in the PSD is *N *= 100, which is in accordance with previous estimates [53]. AMPARs are considered static entities within the PSD, representing a pool of receptors linked to scaffold elements in the PSD. The radius of the PSD is given by *r*_*PSD*_, and the surface area is *A*_*PSD*_. The PSD is placed into the centre of the simulated postsynaptic membrane, the centre of the PSD membrane domain being located at the simulation volume origin of coordinate (0,0,0). The viscosity of the synaptic cleft is chosen such that the diffusion coefficient for glutamate, *D*_*glu*_, matches previous estimates of 0.2 *μ*m^2^/ms [54].

**Table 5:** Kinetic constants. Kinetic constants for AMPAR

Symbol	**Original Value **[37]	Temperature adjusted value (*Q*_10_= 3.0)
*k*_+1_	4.59 * 10^6 ^M^-1^s^-1^	23.85 * 10^6 ^M^-1^s^-1^
*k*_-1_	4.26 * 10^3 ^s^-1^	22.14 * 10^3 ^s^-1^
*k*_+2_	28.4 * 10^6 ^M^-1^s^-1^	147.57 * 10^6 ^M^-1^s^-1^
*k*_-2_	3.26 * 10^3 ^s^-1^	16.94 * 10^3 ^s^-1^
*k*_+3_	1.27 * 10^6 ^M^-1^s^-1^	6.6 * 10^6 ^M^-1^s^-1^
*k*_-3_	45.7 s^-1^	237.46 s^-1^
*α*	4.24 * 10^3 ^s^-1^	22.03 * 10^3 ^s^-1^
*β*	900 s^-1^	4676.54 s^-1^
*α*_1_	2.89 * 10^3 ^s^-1^	15.02 * 10^3 ^s^-1^
*β*_1_	39.2 s^-1^	203.69 s^-1^
*α*_2_	172 s^-1^	893.74 s^-1^
*β*_2_	0.727 s^-1^	3.78 s^-1^
*α*_3_	17.7 s^-1^	91.97 s^-1^
*β*_3_	4.0 s^-1^	20.78 s^-1^
*α*_4_	16.8 s^-1^	87.3 s^-1^
*β*_4_	190.4 s^-1^	989.35 s^-1^

### Simulation execution

Receptor movement in the synaptic spine was simulated using the *Meredys *simulation software (Available at: http://www.ebi.ac.uk/compneursrv/meredys.html). All simulations were run on a Centos 4.2 Linux LSF Cluster. The individual hosts used were a mixture of 32 bit and 64 bit machines. The cluster contains approximately 470 CPU cores across 130 machines. Each run simulated the movement of receptors across the dendritic spine membrane. The parameters for the 'prototypical' reference model are outlined in Table 1. Each change in a parameter from the reference model as indicated in the text was tested with a simulation series. A simulation series consisted of a total of at least 30 individual simulations. The random number generator of the simulation software was seeded with a different values for each simulation. Results obtained were averaged over the number of simulations in a series. Each simulation was run for at least 5 * 10^6 ^iterations, and each iteration had a step length of 1 *μ*s, amounting to a total simulated time of at least 5 s. Output was captured in text files analysed with Perl scripts. The NeuroML input files of the reference model for *Meredys *used for the simulation can be found in the supplementary material.

### Calculation of reaction rates

The *Smoldyn *algorithm used in *Meredys *requires reaction rates to be supplied to in order to determine an appropriate binding radius, *σ *[29]. These rates are calculated from the desired binding radius, the step-length and the diffusion coefficients of the interacting entities by in-house developed software utilising the *Smoldyn *algorithm. Tables 2 &3 show the input rates and the resulting binding radius.

### Determination of MSD plot

The two-dimensional mean squared displacement (MSD) for an ensemble of particles at each time-step was determined as follows:(5)

Where (*x*_*i*_(0), *y*_*i*_(0) is a particles initial position, and (*x*_*i*_(*t*), *y*_*i*_(*t*) is a particles position at time *t*. *N *is the total number of particles and *i *is the particle index.

### Construction of the trace

The trace of a molecule within the membrane was constructed from simulation output file using in-house built software for converting *Meredys *position output information into a trace file. The program takes the position of a particle for successive iteration steps and connects them with straight lines.

## Authors' contributions

DPT ran the simulations, conducted the data analysis, and drafted the manuscript. All authors contributed to the final manuscript. Both authors read and approved the final manuscript.

## Supplementary Material

Additional file 1 **NeuroML input files for receptor incorporation**. The NeuroML input files detailing the receptor incorporation reference model for use with *Meredys*. Click here for file

Additional file 2 **NeuroML input file for glutamate signalling**. The NeuroML input files detailing the glutamates siganlling reference model for use with *Meredys*. Click here for file
